# The Scarface Score: Deciphering Response to DNA Damage Agents in High-Grade Serous Ovarian Cancer—A GEICO Study

**DOI:** 10.3390/cancers15113030

**Published:** 2023-06-01

**Authors:** Antonio Fernández-Serra, Raquel López-Reig, Raúl Márquez, Alejandro Gallego, Luís Miguel de Sande, Alfonso Yubero, Cristina Pérez-Segura, Avinash Ramchandani-Vaswani, María Pilar Barretina-Ginesta, Elsa Mendizábal, Carmen Esteban, Fernando Gálvez, Ana Beatriz Sánchez-Heras, Eva María Guerra-Alía, Lydia Gaba, María Quindós, Isabel Palacio, Jesús Alarcón, Ana Oaknin, Jessica Aliaga, Marta Ramírez-Calvo, Zaida García-Casado, Ignacio Romero, José Antonio López-Guerrero

**Affiliations:** 1 Molecular Biology Lab, Molecular Biology Department, Instituto Valenciano de Oncologia, 46009 Valencia, Spain; 2 Joint IVO-CIPF Cancer Research Unit, 46012 Valencia, Spain; 3 Medical Oncology Department, MD Anderson Cancer Center, 28033 Madrid, Spain; raulmarquez@mdanderson.es; 4 Medical Oncology Department, Hospital Universitario La Paz, 28046 Madrid, Spain; 5 Medical Oncology Department, Hospital Universitario de León, 24008 León, Spain; 6 Medical Oncology Department, Hospital Clínico Universitario Lozano Blesa, 50009 Zaragoza, Spain; 7 Medical Oncology Department, Hospital de Sant Pau i Santa Tecla, 08025 Barcelona, Spain; 8 Medical Oncology Department, Hospital Universitario Insular de Gran Canaria, 35016 Gran Canaria, Spain; 9 Medical Oncology Department, Institut Català d’Oncologia Girona, 17007 Girona, Spain; 10 Medical Oncology Department, Hospital General Universitario Gregorio Marañón, 28007 Madrid, Spain; 11 Medical Oncology Department, Hospital Virgen de la Salud, 45005 Toledo, Spain; 12 Medical Oncology Department, Complejo Hospitalario de Jaén, 23007 Jaén, Spain; 13 Medical Oncology Department, Hospital General Universitario de Elche, 03203 Elche, Spain; 14 Medical Oncology Department, Hospital Universitario Ramón y Cajal, 28034 Madrid, Spain; 15 Medical Oncology Department, Hospital Clínic de Barcelona, 08036 Barcelona, Spain; 16 Medical Oncology Department, Complejo Hospitalario Universitario A Coruña, 15006 A Coruña, Spain; 17 Medical Oncology Department, Hospital Central Asturias, 33011 Oviedo, Spain; 18 Medical Oncology Department, Hospital Universitario Son Espases, 07120 Palma de Mallorca, Spain; 19 Medical Oncology Department, Hospital Universitari Vall d’Hebron, 08035 Barcelona, Spain; 20 Pathology Department, Instituto Valenciano de Oncologia, 46009 Valencia, Spain; 21 Medical Oncology Department, Instituto Valenciano de Oncología, 46010 Valencia, Spain; 22 Department of Pathology, Catholic University of Valencia, 46001 Valencia, Spain

**Keywords:** high-grade serous ovarian cancer, genomic instability, machine learning, PARPi, platinum-based chemotherapy

## Abstract

**Simple Summary:**

The response of high-grade serous ovarian cancer (HGSOC) to DNA-damaging agents largely depends on tumor genomic instability (GI), a phenomenon that affects the entire genome. Nowadays, surrogate biomarkers of this phenomenon, such as *BRCA*-gene mutations, are used in clinical practice to identify patients harboring this characteristic. However, these approaches do not capture the entire picture of GI, mainly due to the lack of information on non-*BRCA* mutation causes and hence, leading to the misclassification of patients. Thus, considering the great interest in studying GI from a comprehensive perspective, this study aims to establish an integrative response-predictive classifier (Scarface Score) for DNA-damaging agents in the context of HGSOC. The Scarface score will support clinical decision-making by correctly selecting the subpopulation of patients with better responses and avoiding overtreatment of those with a low Scarface Score.

**Abstract:**

Genomic Instability (GI) is a transversal phenomenon shared by several tumor types that provide both prognostic and predictive information. In the context of high-grade serous ovarian cancer (HGSOC), response to DNA-damaging agents such as platinum-based and poly(ADP-ribose) polymerase inhibitors (PARPi) has been closely linked to deficiencies in the DNA repair machinery by homologous recombination repair (HRR) and GI. In this study, we have developed the Scarface score, an integrative algorithm based on genomic and transcriptomic data obtained from the NGS analysis of a prospective GEICO cohort of 190 formalin-fixed paraffin-embedded (FFPE) tumor samples from patients diagnosed with HGSOC with a median follow up of 31.03 months (5.87–159.27 months). In the first step, three single-source models, including the SNP-based model (accuracy = 0.8077), analyzing 8 SNPs distributed along the genome; the GI-based model (accuracy = 0.9038) interrogating 28 parameters of GI; and the HTG-based model (accuracy = 0.8077), evaluating the expression of 7 genes related with tumor biology; were proved to predict response. Then, an ensemble model called the Scarface score was found to predict response to DNA-damaging agents with an accuracy of 0.9615 and a kappa index of 0.9128 (*p* < 0.0001). The Scarface Score approaches the routine establishment of GI in the clinical setting, enabling its incorporation as a predictive and prognostic tool in the management of HGSOC.

## 1. Introduction

The term ‘genomic instability’ (GI) describes the characteristic of cells to progressively accumulate genomic alterations. In recent years, because of its increasing importance in the field of oncology, GI has gained greater attention in translational research [1]. GI is a hallmark of cancer and is relevant not only as an intrinsic feature of tumor cells but also as a potential driving force of tumorigenesis [2]. Although GI is present in every cancer type, some tumors show a remarkable accumulation of alterations [3]. High-grade serous ovarian cancer (HGSOC) is of particular interest in this respect. HGSOC is a molecularly and clinically heterogeneous disease that is characterized by TP53 mutations and DNA damage homologous recombination repair (HRR) deficiency (HRD) in approximately 50% of patients [4]. Deficiencies in this pathway could have different molecular causes in addition to classically known *BRCA1/2* mutation, such as other HRR-genes mutations and epigenetic modifications [5]. The HRD phenotype represents a clear molecular subtype that is highly enriched in copy number alteration patterns, which play important roles in oncogenesis, progression, and metastasis [2,6]. The so-called HRD phenotype is defined as a clinical profile similar to tumors harboring BRCA gene alterations. That is, showing a higher progression-free survival treated mainly with platinum salts and PARP inhibitors, among other therapies [7]. Copy number alteration patterns can be classified by the presence of specific GI events, also called genomic scars, reflecting a loss of genome integrity [8]. These genomic scars may be reliable biomarkers for homologous recombination repair deficiency (HRD) and could potentially be used to identify patients who would benefit from specific types of anticancer therapies, such as platinum-based chemotherapies or poly(ADP-ribose) polymerase inhibitor (PARPi) therapy [9,10,11]—the clinical utility of which has been shown in several clinical trials, including PAOLA [12], PRIMA [13], VELIA [14] and ATHENA [15]. As such, GI is a potential predictive and prognostic biomarker [6]. Because of these clinical implications, researchers are attempting to define GI status in order to select patients who will benefit from these therapeutic approaches.

Classically, the determination of HRD status has relied on *BRCA1* and *BRCA2* genotyping [16], but the HRR pathway involves a vast range of proteins, most of which are reportedly mutated in tumor samples [17]. Today, the development of high-throughput techniques allows the integrative analysis of multiomic data to generate machine learning models, which can more comprehensively determine HRD status [18,19].

Based on the above, the aim of this study was to develop a methodologic and analytic approach to determining GI status in patients with HGSOC using a comprehensive strategy that integrates data from single-nucleotide variations, somatic copy number alterations, and transcriptomics. These data were used to build a model (the Scarface score) that could predict a patient’s response to DNA-damaging agents.

## 2. Materials and Methods

### 2.1. Patient Selection

The study used 190 formalin-fixed and paraffin-embedded (FFPE) HGSOC samples that were ambispectively collected from patients treated at multiple centers from 2007 to 2020 (BorNeO 1703). An ambispective study implies the combination of both retrospective and prospective data, including past, present, and future time points. All patients signed an informed consent form approved by the required ethics committees, and the study was approved by the ethics committee of Fundación Instituto Valenciano de Oncología in 2021 (LBM-02-20, SCARFACE). The informed consent of patients was obtained following institutional, ethical, and legal regulations. The inclusion criteria were age ≥18 years at inclusion, diagnosis with HGSOC, and previous first-line treatment with platinum-based chemotherapy.

### 2.2. Mutational and Copy Number Variants Analysis

DNA extraction was performed using three 20 μm-thick sections of FFPE tumor blocks and a QIAamp DNA FFPE tissue kit (Qiagen, Hilden, Germany). The final concentration was measured spectrophotometrically using NanoDrop ND-1000 (Eppendorf, Hamburg, Germany). Genomic concentration, DNA integrity, and fragment size were determined by using a TapeStation 4200 bioanalyzer (Agilent, Santa Clara, CA, USA).

Libraries were prepared using the SureSelectXT HS Target Enrichment Kit using the Magnis NGS Prep System (Agilent, Santa Clara, CA, USA). Briefly, 200 ng of extracted DNA was enzymatically fragmented to a size range of 150–200 base pairs. Each library was then hybridized with a SureSelectXT HS custom panel combined with Agilent OneSeq backbone 1 Mb according to the manufacturer’s protocol. The custom panel analyzed the following DNA damage response genes: *BRCA1*, *BRCA2*, *BARD1*, *BRIP1*, *CHEK1*, *CHEK2*, *FAM175A*, *NBN*, *PALB2*, *ATM*, *MRE11A*, *RAD51B*, *RAD51C*, *RAD51D*, *RAD54L*, *FANCI*, *FANCM*, *FANCA*, *ERCC1*, *ERCC2*, *ERCC6*, *REQL*, *XRCC4*, *HELQ*, *SLX4*, *WRN*, *ATR*, *PTEN*, *CCNE1*, *EMSY*, *TP53*, *MLH1*, *MSH2*, *MSH6*, and *PMS2*.

Although HRR genes were overrepresented in the panel, genes belonging to the base excision repair, nucleotide excision repair, and mismatch repair pathways were also incorporated into the design. The OneSeq backbone was used to obtain copy number variants (CNVs), consisting of 147,000 single-nucleotide polymorphisms (SNPs) homogeneously distributed along the genome. Pooled libraries were sequenced (100 bp paired-end) using the NextSeq 550 System (Illumina, San Diego, CA, USA). A secondary analysis was performed using HaplotypeCaller (Broad Institute, Cambridge, MA, USA) for variant calling and VariantStudio 4.0 for annotation (Illumina). Variants were selected after a filtering process based on the following analytical parameters: coverage >100× (covered in forward and reverse sense); allele frequency >5%; and annotation of Pathogenic, likely pathogenic, or VUS with a prediction of pathogenicity with Varsome classifier. Germline *BRCA1/2* alterations were obtained from analyses carried out at each hospital of origin.

Bioinformatics analysis to obtain copy number events was performed using an in-house pipeline based on the CNVkit algorithm [20]. This pipeline was internally customized to ensure the suitability and reliability of the method (Supplementary Data S1 and Figures S1–S5). The CNVkit algorithm uses sequencing data from target and anti-target regions to infer copy number status. Circular binary segmentation was chosen for the segmentation step. The variant calling step was performed using Mutect2 (Broad Institute). Normalization was applied by using median read counts from a set of 10 control samples from healthy peritumoral ovarian tissue.

Independently, the panelcn.MOPS package (version 1.17.1) [21] was used to evaluate copy number changes at the gene level—particularly *CCNE1* amplification.

The presence of HRD-associated genomic scars (loss of heterozygosity (LOH), large-scale transitions, number of telomeric allelic imbalances, and a combined score (HRD score)) was assessed using the scarHRD package (version 0.1.1) for R [22].

The parameter settings and codes used for GI determination with CNVkit software and the script to extract analytical features are available at https://github.com/afernandezse/Pola_Phase2_GI_traslational (accessed 22 January 2022).

### 2.3. Transcriptomic Analysis

Gene expression analysis was performed using the HTG EdgeSeq System (HTG Molecular Diagnostics, Tucson, AZ, USA). This technique is based on RNA sequencing consisting of a prehybridization step with specific probes using a quantitative nuclease protection assay, followed by a standard next-generation sequencing (NGS) protocol. This technique requires a small input (i.e., 5 μm FFPE section and an area of 15 mm^2^). The panel focuses on a selection of 2549 oncology-related mRNAs (the Oncology Biomarker Panel) rather than analyzing the entire transcriptome, obtaining the appropriate dynamic range in gene expression analysis. Gene expression data were parsed using HTG EdgeSeq Parser version 5.3.0.7184. Quality control was performed using HTG Reveal version 3.0 (HTG Molecular Diagnostics). Raw read counts were normalized according to the median [23].

### 2.4. Model Fitting

To improve the current detection of HRD-related GI, a data-mining model integrating several biological approaches was proposed. The model included genomic and transcriptomic data from 190 HGSOC samples, from which all data were available for 183 samples. The first layer of the model comprised 147,000 SNPs uniformly distributed along the entire genome at a resolution of 1 Mb. The second layer, comprised of GI parameters, was derived from CNVkit results. Finally, gene expression data obtained from targeted RNA sequencing of 2549 genes was the third layer. Because of the high number of SNP parameters, those that were less informative were removed under the criteria of a low number or near zero variance in total counts per SNP.

Briefly, the model fitting on the first and third layers consisted of three parts. First, feature selection was performed by extracting attributes using the ANOVA test, the signal-to-noise ratio, significant parameters identified from logistic regression analysis, recursive feature extraction [24], and the Boruta algorithm [25]. Second, model feeding was conducted. Each resulting set of features was tested to build three data-mining models using the following algorithms: support vector machine, random forest, and neural network (Supplementary Data S2). Third, specific hyperparameters were tuned. Second-layer building followed the same procedure but without feature extraction.

The final model consisted of an ensemble model (which was termed the Scarface score), in which the best-performing data-mining model was fed with its paired selected parameters. This model was benchmarked by studying its mean accuracy and kappa index from 500 bootstrapping iterations (detailed in Supplementary Data S2). Each model, including the ensemble model (the Scarface score), was trained and validated using two series, which were randomly selected from the total 183 HGSOC samples in a proportion of 70/30, respectively. The models were trained to discriminate between patients with a response to platinum-based chemotherapy ≥12 months (responders) versus <12 months (non-responders).

### 2.5. Statistical Analysis

The chi-square and Fisher’s exact tests were used to compare categorical GI and clinical and pathological variables. Non-parametric Wilcoxon and Kruskal–Wallis tests were used for continuous variables.

For time-to-event variables, survival analysis was performed using Kaplan–Meier estimation, and significance was obtained by log-rank testing. Univariate and multivariate Cox regression was also performed. Statistical significance was considered at *p* < 0.05. All tests were two-tailed. The time-to-event variables investigated were platinum-free interval (PFI), defined as the time between the end of platinum-based chemotherapy and relapse; progression-free survival (PFS) to PARPi, defined as the time between the start of PARPi treatment and disease progression; and overall survival (OS), defined as the time between diagnosis and death. The performance of the models was evaluated using the ROCR and pROC packages from R version 4.1.2. Statistical analyses were performed using R studio version 2021.09.0.

## 3. Results

### 3.1. Study Population

FFPE tumor blocks from 190 patients with HGSOC were analyzed. Clinical parameters of the patient population are shown in Table 1. The median follow-up of the studied population was 31.03 months (range 5.87–159.27 months). Median PFI after first-line therapy was 16.28 months (range 0–83.33 months), the recurrence rate after first-line therapy was 52.11% (99/190), and the median PFS to PARPi was 11.03 months (range 1.03–64.63 months). Overall, 20.53% of patients had died at the time of data analysis.

### 3.2. Mutational Distribution and Clinical Implications

**Figure 1:**
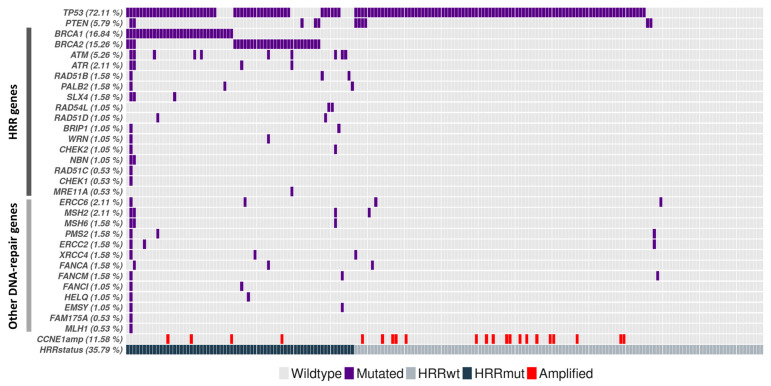
Distribution of mutations in DNA damage repair genes among 190 patients with high-grade serous ovarian cancer, stratified by HRR gene status. HRR, homologous recombination repair; mut, mutated; wt, wild-type.

Mutational analysis was performed based on the results of the NGS custom panel, which analyzed 35 DNA damage repair genes. As expected, the most frequently mutated gene was *TP53*, which was mutated in 72.11% (137/190) of samples, followed by *BRCA1* and *BRCA2*, with incidences of 16.84% (32/190) and 15.26% (29/190), respectively. Germline mutations were detected in 59.02% (36/61) of patients with *BRCA1/2*-mutated HGSOC. Other HRR genes were also found to be altered, with a total incidence of 11.05% (21/190), some of them coexisting with *BRCA* mutations. In addition, alterations in other DNA damage repair genes were also identified (Figure 1). Mutational data were used to classify tumors as HRR-proficient or HRD, according to the mutational status of pathway-specific genes. Hence, 35.79% (68/190) of patients were considered HRR mutated (HRRmut).

**Figure 2:**
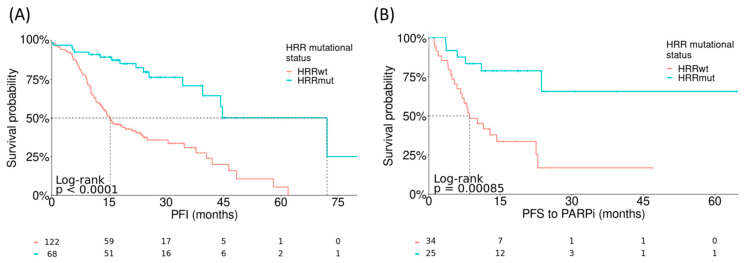
Log-rank test to evaluate the predictive ability of an HRR gene mutation-based classifier with respect to (**A**) PFI, HR = 0.25 (95% CI: 0.15–0.43) and (**B**) PFS to PARPi therapy, HR = 0.25 (95% CI: 0.1–0.62). HRR, homologous recombination repair; mut, mutated; PARPi, poly(ADP-ribose) polymerase inhibitor; PFI, platinum-free interval; PFS, progression-free survival; wt, wild-type.

Non-parametric and log-rank tests were used to evaluate the ability of HRR mutation status to predict response to DNA-damaging drugs (including platinum-based and PARPi therapies). The results revealed differences for tumors HRR wildtype (HRRwt) versus HRRmut with respect to both PFI (*p* = 5 × 10^−8^), with a median PFI of 15.3 and 72.1 months, and PFS to PARPi (*p* = 0.00085), with a median of 8.53 months for HRRwt and were not achieved by HRRmut, demonstrating the prognostic impact of HRR mutation status (Figure 2).

CCNE1 has previously been implicated in the prognosis of patients with HGSOC [4], and therefore, the addition of the CCNE1 amplified cases in this series could increase the accuracy when classifying patients. For that reason, CCNE1 amplification was evaluated in silico in this series. Patients whose tumors harbored amplifications in CCNE1 (22/190, 11.58%) were classified as an independent subgroup to evaluate the prognostic implication of each genomic alteration. The addition of *CCNE1* amplified cases as a new independent group showed significant differences in the log-rank tests for both PFI (*p* < 0.0001) and PFS to PARPi (*p* = 0.00012) (Figure S6). In the case of PFS to PARPi, the presence of *CCNE1* amplification was associated with the worst-prognosis group, followed by HRRwt and, finally, HRRmut.

### 3.3. Copy Number Parameters and Their Clinical Implications

The applied NGS approach also includes 147,000 SNPs homogeneously distributed among the whole genome. These data facilitated the assessment of GI based on copy number analysis by using an in-house pipeline. Hence, we were able to establish GI profiles and quantify them using different predefined parameters (Supplementary Data S3). Each GI parameter was tested for associations with continuous and categorical response variables. GI parameters that were more significantly associated with PFI in non-parametric tests were the total number of LOH events of >15 Mb (*p* = 0.019) and the percentage of the genome that was altered by LOH of >15 Mb (*p* = 0.016) (Figure S7). However, there were also other GI parameters also resulted in significant correlation, as specified in Supplementary File.

The correlation between pre-established HRD scores, as previously described [26], and response variables was also evaluated. The highest significance for predicting PFI was seen with the LOH parameter stratified by its median value (*p* = 0.0071), followed by the HRD score stratified by its median value (*p* = 0.031). However, none of the pre-established HRD scores investigated was able to significantly predict PFS to PARPi (Figures S8 and S9).

Aiming to optimize the generated data, even though GI parameters on their own could work as a predictive biomarker and to improve the currently available biomarkers, the combination of them was used as a base to build a predictive model.

Finally, GI profiles, described by the presence of GI parameters, were determined to compare the different HRR mutational-based populations. As expected, a higher accumulation of GI was found in samples harboring mutations in the HRR pathway and was especially enriched for those with *BRCA* mutations (Figure S10).

### 3.4. Independent Model Fitting and Building of the Integrative Ensemble Model (Scarface Score)

In order to adjust a machine learning strategy to predict response to platinum-derived therapy, attributes from three different sources were used. The first model was derived from the raw coverage information of 147,000 SNPs, while the third model contained gene expression data from 2549 genes obtained from targeted RNA sequencing results. Feature selection was performed using several strategies, as described in the Materials and Methods. The second model included the most representative parameters of the GI phenomenon but was not subjected to feature selection because of a low number of features. Each set of selected parameters was tested and coupled with a data-mining algorithm. Every possible combination of the data-mining algorithm and selected features was tested.

The best performances were seen with a support vector machine with eight SNPs (‘SNP model’; Table S1), a support vector machine with 28 GI parameters (‘GI model’), and a neural network with the expression of seven genes (‘HTG model’; Table S2). Selected features of each model are described in Supplementary Data S3. The performance of each model is shown in Table 2. Weights and main characteristics of the features included in each of the three models and the ensemble are included in Tables S3–S6. Among the three single-source models, the best performance was obtained with the GI model, which had an accuracy of 0.9038. Finally, an ensemble model (the Scarface score) was developed based on a support vector machine algorithm, using as an input the 43 attributes from the individual models described above. The ensemble model was trained with a bootstrapping of 500 iterations and obtained an accuracy of 0.96 and a kappa index of 0.91, outperforming all three single-source models. All performance parameters were obtained from the validation series.

**Figure 3:**
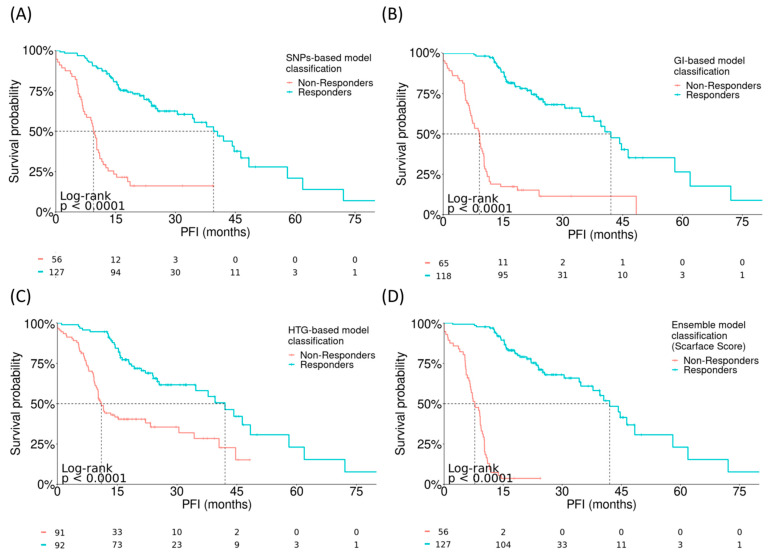
Correlation between the fitted models and PFI. Log-rank tests evaluating the performance of (**A**) SNP-based model, HR = 0.19 (0.12–0.29), (**B**) GI-based model, HR = 0.12 (95% CI: 0.08–0.19), (**C**) HTG-based model, HR = 0.34 (95% CI: 0.22–0.51), and (**D**) integrative ensemble model (Scarface score), HR = 0.046 (95% CI: 0.027–0.077). GI, genomic instability; PFI, platinum-free interval; SNP, single-nucleotide polymorphism.

The clinical impact of each model was tested in the whole population of patients with HGSOC (n = 183) by using a log-rank test with PFI as a time-to-event variable. All four models, including the ensemble model, were able to distinguish responders from non-responders with significant differences in PFI (all *p* < 0.0001; Figure 3). The HTG-based model was found to be the most limited, while the highest statistical significance was obtained using the ensemble model (*p* < 2 × 10^−16^).

**Figure 4:**
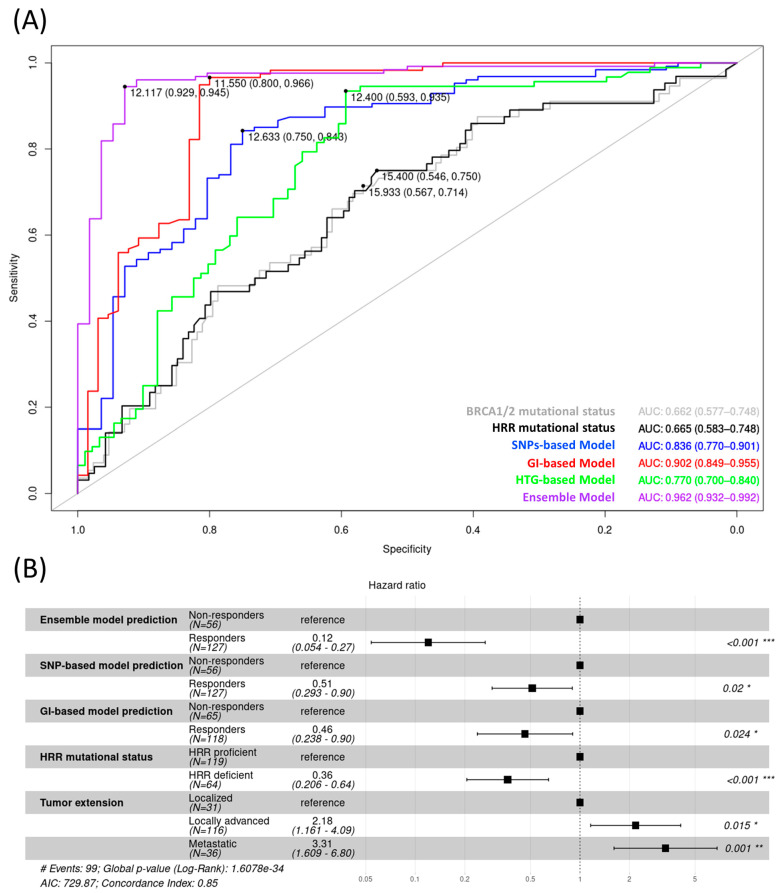
Performance of the predictive models. (**A**) ROC curves comparing the three predictive models, the ensemble model, and HRR-based classifications as categorical variables, with PFI as a continuous variable. (**B**) Multivariate Cox regression analysis for HRR mutation status, tumor extension, and the performance of the models. Tumor extension was stratified based on stage: localized (I–IIB), locally advanced (III–IVA), or metastatic (IVB) regarding PFI. * *p*-value ≤ 0.05, ** *p*-value < 0.01 and *** *p*-value < 0.001. GI, genomic instability; HRR, homologous recombination repair; PFI, platinum-free interval; SNP, single-nucleotide polymorphism. # Characteristics of the regression.

The goodness-of-fit of each model was evaluated using receiver operating characteristic (ROC)curves, which showed how well each predictive model discriminated between patients with a PFI ≥12 versus <12 months. As expected, the highest discriminative power was obtained with the ensemble model, which had an area under the curve of 0.962, a sensitivity of 0.929, and a specificity of 0.945 (Figure 4A).

**Figure 5:**
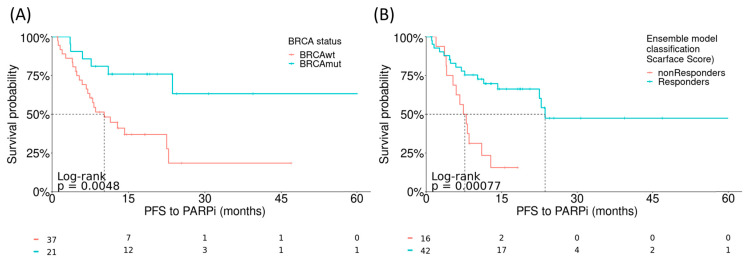
Correlation between the fitted models and PFS to PARPi. Log-rank tests evaluating the performance of: (**A**) *BRCA* mutation-based classification and (**B**) the integrative ensemble model (Scarface score). PARPi, poly(ADP-ribose) polymerase inhibitor; PFS, progression-free survival.

Although the algorithms were trained to predict response to platinum-based chemotherapy, the ultimate aim of the study was to develop a model that could identify patients who are candidates for PARPi therapies. Thus, the ability of the models to discriminate the best responders to PARPi therapies was also investigated using log-rank testing in a sub-cohort of 58 patients from the overall population who had received PARPi therapy in addition to first-line platinum-based chemotherapy. The performance of the models was compared with the stratification based on *BRCA* mutation, which is the current gold standard for selecting patients to receive PARPi therapy. The ensemble model was found to have a *p*-value of 0.00077 for non-responders versus responders, which outperformed *BRCA*-based classification (*p* = 0.0048) (Figure 5 and Figure S11), thus improving the discriminant power of the gold standard.

The ability of the models to predict overall survival was also evaluated. All models reached statistical significance, with the greatest significance seen for the ensemble model (Figure S12C–F). In contrast, classification based on *BRCA* or HRR gene status appeared unable to significantly predict overall survival (Figure S12A,B). Exact *p*-values and summarized survival analyses are shown in Table S7.

In addition to model performance, a multivariate analysis was performed to evaluate the ability of different clinicopathologic and mutational parameters to stratify patients according to overall survival. The most discriminant parameter was the ensemble model prediction (hazard ratio (HR) 0.12). However, other parameters, such as tumor extension (locally advanced, HR 2.18; metastatic, HR 3.31 and HRR mutation status (HR 0.36), also contributed to risk assessment (Figure 4B). Additional Cox analyses were performed evaluating a higher number of variables (Figure S13).

## 4. Discussion

GI, as a surrogate of HRD, has risen as a prognostic and predictive tool in HGSOC [27]. While HRR-based stratification, based on any alteration or effect in the genome, is widely recognized as essential, many efforts have been made to develop and clinically validate academic tools based on different approaches [28,29,30]. In this study, we developed three single-source models based on SNPs, GI, and RNA expression analysis, respectively, and an integrative ensemble model (the Scarface score) to predict response to DNA-damaging agents—particularly platinum-based chemotherapy and PARPis. The Scarface model—which combined eight SNPs, 28 GI parameters, and the expression of seven genes—showed the best performance, with an accuracy of 0.9615 and a kappa index of 0.9128 in the validation series. However, the single-source models could also be suitable and efficient tools in a real-life clinical setting, helping to guide the clinical management of patients. The proposed models were built based on three layers: SNP deep NGS, a CNV profile using in silico algorithms, and targeted RNA sequencing using HTG EdgeSeq technology. Each layer has its strengths and limitations, but ultimately, each underpins the others. This design accounts for the different mechanisms by which HRD is produced and tries to mimic the complex biological context (e.g., genomic, transcriptomic). These different levels of biological information could be better represented by a multiomic approach. For this purpose, the capacity of machine learning to account for complex interactions in large datasets [31] made it optimal for the study of GI based on drug response. Several machine learning models (support vector machine, random forest, neural network, decision tree, and naïve Bayes) were adjusted with different parameters and hyperparameters, and the resulting models were benchmarked to rank the best performance for each layer.

Commercial solutions, such as MyChoice^®^ CDx Plus (Myriad Genetics, Salt Lake City, UT, USA) and the FoundationOne^®^ CDx (Foundation Medicine, Cambridge, MA, USA), which are based on identifying genomic scars, HRR gene mutations and LOH, have already shown their clinical benefit in clinical trials [32,33,34]. However, even if each model succeeds in predicting *BRCA1/2* status (for which they are trained), the fact that they do not cover other molecular mechanisms (e.g., CNV or gene expression) means that they do not provide information on other HRD-causing mechanisms independent of *BRCA* gene status [35,36]. In addition, there has not yet been a direct prospective comparison between the two tests. One study reported on the interchangeability of the MyChoice assay using LOH alone compared with the GI score (GIS) and showed poor agreement; among 3209 wild-type *BRCA* genes, 53% of those assigned as unstable by GIS (cut-off ≥ 33) were assigned as HRD-negative by %LOH criteria, while only 4% of unstable tumors assessed by %LOH were positive using GIS. Considering *BRCA1/2* and the official GIS cut-off of ≥42, an agreement was 64.9% for positive cases and 96.6% for negative cases [37]. Similar discrepancies were also seen in a retrospective analysis that found that 23% of samples were classified as GI stable, with an LOH percentage of <16%, by FoundationOne harbored *BRCA1/2* germline mutations [38]. These facts, together with the high costs of these tests and long turnaround times for results, constitute the main limitations of both commercial tests.

With the Scarface model, we have integrated GI parameters—equivalent to HRD status—rather than HRR mutations to differentiate patients more accurately according to PFI. Information about gene expression is also provided, supporting the GI and contributing to responder–phenotype processes. This approach has the advantage of studying the GI phenomenon as a whole: at the genomic, chromosomal, and transcriptomic levels. Due to the impossibility of comparing our data with the gold standard, such as those mentioned above, since patients included in the study lack this type of determination, we compared the model with a classification based on HRR gene mutations (*BRCA1/2* only and all HRR genes) and scores from the scarHRD pipeline [22]. In our series, 35.8% of samples had HRR gene mutations, with *BRCA1* and *BRCA2* mutations in 16.84% and 15.26%, respectively. Additionally, amplification of *CCNE1* was performed in our series, with an incidence of approximately 12%. Co-occurrence of BRCA1/2 mutations and CCNE1 amplification were found in approximately 7% of BRCA1/2 mutated cases, similar to the frequencies found in the OC-TCGA [4]. Even though these alterations are found together in a very low number of cases, there are not mutually exclusive. Those samples harboring mutations in HRR genes were classified as HRD for comparison. Both stratifications—based on *BRCA1/2* mutation and all HRR genes—were able to identify patients who would have an extended PFI (both *p* < 0.0001) and PFS to PARPi (*BRCA1/2*, *p* = 0.0048; all HRR genes, *p* = 0.0013). In this particular case, adding other HRR genes to *BRCA1/2* when classifying patients improved statistical power and increased the prognostic and predictive value. However, as recently reported, they do not always overlap GI, suggesting higher accuracy of the GI score over an HRR gene panel to define an HRD phenotype [39,40]. For that reason, approaches at different levels, such as genomic scars, are gaining strength in the assessment of GI.

The scarHRD pipeline was applied to compare the performance of the classifiers. This pipeline has been trained to identify the genomic scars evaluated by the validated commercial solutions, LOH, large-scale transitions, telomeric allelic imbalances, and HRDscore. However, the results were not as good as expected. Differences in methodologic and analytic procedures caused a loss of statistical significance when analyzing our series, with several potential causes. First, in this approach, GI data were derived from NGS data covering a backbone and a medium-size panel, whereas the MyChoice kit was validated and calibrated using a comparative genomic hybridization array. Second, the CNVkit method was used with the parameters specifically tuned to our clinical scenario, including pre-analytical factors such as tumor burden in the sample. The best results were obtained when the series was stratified based on the median number of LOH events (PFI, *p* = 0.0071; PFS to PARPi, *p* = 0.07) and median HRD score (PFI, *p* = 0.031; PFS to PARPi, *p* = 0.28), but significance was only reached for PFI and not for PFS to PARPi. In contrast, the Scarface model achieved the highest statistical significance for both PFI (*p* < 2 × 10^−16^) and PFS to PARPi *(p* = 0.00077), improving the predictive performance above that of previously used classifiers.

As mentioned, the predictive algorithm was trained and validated in an ambispective, multicentric, real-life cohort of patients with HGSOC using PFI as an endpoint. Because of the real-life design, information regarding PFS to PARPi was not as accurate as expected; PFS to PARPi data were collected with respect to different lines of therapy (first-line therapy in 23 patients and second or later lines in 35 patients), different treatment combinations and schemes, and different PARPi drugs. As such, PFS to PARPi was not a suitable parameter for training and validating the model. The real-world nature of the series, which lacks centralized review, probably implies the misclassification of some studied cases. The concordance between the centralized review and the first diagnosis is approximately 70%, as previously presented in other works in OC [41]. This could be the cause of the low number of TP53 alterations found (72% in this cohort vs. more than 90% in other series [4]). The same cause could be responsible for the high number of *BRCA1/2* mutated cases without *TP53* alteration, uncommonly found in HGSOC. Representation of other histologies with different mutational patterns, such as the case of endometrial OC [42], could be influencing the results. Even if this fact constitutes a limitation of the study, it is also presented as a strength since it represents the reality of the clinical practice in which the model would be potentially used. Otherwise, another limitation of the study consists of the fact that the data sources are quite specific; thus, it is necessary to sequence the samples with the kit described in material and methods containing a backbone. Additionally, this fact limits the availability of data in public repositories. Therefore, although the presented algorithm showed that HRR mutations had predictive value for PFS to PARPi, the model should be further evaluated in a cohort with homogeneous PARPi response data to validate its clinical benefit. In addition, because this model addresses GI from different levels of regulation, it seems that it would be plausible to calibrate the model to predict response with different cut-offs in other tumors in which GI may play an important role in response to therapy, such as advanced prostate cancer with *BRCA* mutations or pancreatic cancer. Analogously, new optimal cut-offs for GIS and genomic LOH have been proposed in the VELIA and ARIEL2 clinical trials [5,39]. Thus, there is room for improvement in the exposed GI study approach.

## 5. Conclusions

The Scarface score constitutes a useful academic tool to predict response to DNA-damaging agents in HGSOC and, potentially, in other HRR-deficient tumors. This algorithm addresses the limitations of available and validated commercial solutions by looking at GI and the molecular biology of the tumor from a more comprehensive point of view.

## Figures and Tables

**Table 1:** Main clinical, pathological, and treatment-related variables of the whole series.

Clinical Parameter		N	%	Clinical Parameter		N	%
Histology	High-grade serous ovarian cancer	190	100	Surgery	Yes	167	87.9
Stage	IA	7	3.7		No	23	12.1
	IC1	6	3.2	Primary debulking surgery	Yes	114	68.3
	IC2	9	4.7		No	53	31.7
	IIA	4	2.1	Residual disease after primary debulking surgery	Yes	18	15.8
	IIB	6	3.2		No	96	84.2
	IIIA1	8	4.2	First-line platinum therapy	All	190	100.0
	IIIA2	5	2.6	Relapse after first-line therapy	Yes	99	52.1
	IIIB	10	5.3		No	91	47.9
	IIIC	77	40.5	Received PARP	Yes	59	31.1
	IVA	12	6.3		No	131	68.9
	IVB	27	14.2	Progression with PARPi	Yes	29	49.1
					No	30	50.9
	NA	19	10.0	Exitus	Yes	39	20.5
					No	151	79.5
Stage (aggregated)	Localized (I-IIB)	34	17.9				
				Clinical parameter	Median (range)		
	Locally Advanced (III-IVA)	120	63.2	Age at diagnosis, years	59.2		
					[34.1–83.9]		
	Metastatic (IVB)	36	18.9	Platinum-free interval, months	16.3		
					[0.0–83.3]		
Type of biopsy	Excisional	132	69.5	PFS to PARPi therapy, months	11.0		
	Incisional	35	18.4		[1.0–64.6]		
	Tru-Cut	23	12.1	Follow-up, months	31.0		
*BRCAg*	WT or benign/Likely benign	141	71.2		[5.9–159.3]		
	Variant of unknown significance	13	6.8	Overall survival, months	31.0		
	Pathogenic	36	18.9		[5.87–159.27]		

PARPi, poly(ADP-ribose) polymerase inhibitor. NA, not available.

**Table 2:** Performance of the different predictive algorithms tested.

Model	TP/TN/FP/FN	Accuracy (95% CI)	Sensitivity	Specificity	Kappa
SNP model	29/13/5/5	0.8077 (0.6747–0.9037)	0.7222	0.8529	0.5752
HTG model	25/17/1/9	0.8077 (0.6747–0.9037)	0.9444	0.7353	0.6154
GI model	31/16/2/3	0.9038 (0.7897–0.968)	0.8889	0.9118	0.7903
Ensemble model	34/16/2/0	0.9615 (0.8679–0.9953)	0.8889	1.0000	0.9128

FP, false positive; FN, false negative; GI, genetic instability; SNP, single nucleotide polymorphism; TP, true positive; TN, true negative.

## Data Availability

The datasets used and/or analyzed during the current study are available from the corresponding author upon reasonable request.

## Supplementary Materials

The following supporting information can be downloaded at: https://www.mdpi.com/article/10.3390/cancers15113030/s1, Figure S1: Differences in GI parameters according segmentation used in CNVkit pipeline; Figure S2: Differences in GI parameters adjusting *p*-value in CNVkit pipeline; Figure S3: Differences in GI parameters according tumor burden in CNVkit pipeline; Figure S4: GI parameters according to pre-filtering step in saasCNV pipeline; Figure S5: Comparison of GI parameters between implemented pipe-lines, highlighting differences for the assessment of each feature; Figure S6: Log-Rank test to evaluate the predictive information of HRR-gene mutation and CCNE1 amplification-based classification regarding (A) PFI and (B) PFS to PARPi; Figure S7: Correlation of GI parameters with PFI assessed by non-parametric tests; Figure S8: Log-Rank tests evaluating the implication of predefined HRD scars parameters from scarHRD package in correlation with PFI and PARPi response; Figure S9: Correlation of HRD score obtained on scarHRD package and time-to-event variables; Figure S10: Distribution of total number of LOH events > 15 Mb between BRCA-based populations; Figure S11: Log-Rank tests evaluating the implication of predictive models with PARPi response; Figure S12: Log-Rank tests evaluating the implication of mutational-based classifiers and predictive models with OS; Figure S13: Multivariate analysis performed by Cox regression for clinicopahological parameters, HRR alteration and three-source mod-el performance in addition to ensemble model; Table S1: Selected parameters SNPs model; Table S2: Selected parameters HTG model; Table S3: Weights and main characteristics of the parameters includ-ed in the layer 1 of SNPs deep sequencing; Table S4: Weights and main characteristics of the parameters includ-ed in the layer 2 of Genomic Instability related parameters; Table S5: Weights and main characteristics of the parameters includ-ed in the layer 3 of gene expression; Table S6: Weights and main characteristics of the parameters includ-ed in the lensemble scarface model; Table S7: Log-rank test results for single-source and ensemble mod-el. Additionally, PFI, BRCA (germline and somatic mutations) and HRR-based (all HR-genes interrogated in the panel, including BRCA1/2) classifications were added; Data S1: CN pipelines: personalization of working parameters; Data S2: Methods model fitting; Data S3: Selected parameters.
