# Supplementary material for: The Scarface Score: Deciphering Response to DNA Damage Agents in High-Grade Serous Ovarian Cancer—A GEICO Study
Source: Cancers (Basel). 2023 Jun 1;15(11):3030. doi: 10.3390/cancers15113030 (PMC10252660; doi:10.3390/cancers15113030)
Supplement: Supplementary file 1 [file cancers-15-03030-s001.zip › cancers-2384090-supplementary.pdf]

Supplementary data S1.-CN pipelines: personalization of working parameters

CNA analysis was performed by using three different pipelines: CNVkit, saasCNV and SureCall. To calibrate the threshold levels among the numerous CNA-derived parameters comparisons at distinct levels were performed. While saasCNV and SureCall are limited to slight personalization, CNVkit has a greater number of analytical parameters. In this case, segmentation pipeline (CBS or FLASSO), p-value cut-off, personalized or pre-established tumor content of the sample and post-analytical filtering were evaluated. Firstly, Segmentation pipelines were evaluated. Differences basically appeared in the total number of CN events, higher in CNVkit, while presenting a lower median length. However, the total percentage of altered genome was not significantly different (Figure S1). The selected segmentation pipeline was CBS, due to higher reliability of events of greater length and the suitability to perform the subsequent analysis to call LOH events.

A tuning of CNVkit pipeline parameters was performed as follows: A most restrictive p-value in the Segmentation algorithm was assayed (p=0.001), in this scenario, fragments of greater length are built by the Segmentation algorithm (p=0.00035). The selection of these events caused no differences regarding total number of events while

the percentage of altered genome was significantly higher (p=0.00036) (Figure S2). Using a restrictive filter does not imply losing CNA information while obtaining more reliable results, so that, a p-value of 0.001 was chosen for the subsequent analysis.

The tumor content was evaluated to know whether it was decisive in the analysis of CNA. To do that, establishment of values of 50, 80 and 100 % of tumor content for the same sample were applied during the analysis. Results showed significant differences when applying different tumoral content in total number of events (p=0.00046) and a tendency in percentage of tumor altered and CN length (Figure S3). Hence, establishing and applying its own tumor content per each case will be essential for the following analysis.

Finally, the post-analytical filter was slightly different. Filter based on median or quartile CNVkit assigned weight of events was applied after the analysis, followed by another filter by 1 Mb in length (Figure S4). The application of the filtering step before that, resulted in no differences due to the median and 1st quartile values being both below 1 Mb. However, considering the high number of total events obtained and trying to simplify the following analysis, above-median filter after the 1 Mb length filter was applied.

On the contrary, saasCNV and SureCall were easily adjusted to the nature of the data. For saasCNV, it was only evaluated the pre-

filtering step during the analytical process. In regards to that, the total number of events was considerably lower when not applying pre-filtering steps. Then, the decision was to perform this step, looking for more reliable and handle results. On the other hand, SureCall was applied using the default parameters and chosen healthy control of the performed data.

Circular Binary Segmentation (CBS) was applied for the final selection of parameters and to incorporate: true tumor content from the sample, instead of its estimation based on sample ploidy, the use of a more astringent p-value of 0.001 and the filtering of results by median weight and fragment size, selecting those shorter than 1 Mb to increase specificity in the CNA calling when using CNVkit. SaasCNV adjustment was limited to perform a prefiltering step whereas SureCall was not customized at all.

Once the pipelines were fitted, the ability of each pipeline to assess GI was evaluated. Firstly, values of different GI parameters obtained from the studied pipelines were compared. Significant differences appeared in all performed comparisons, mainly showing a higher percentage of altered genome in SureCall results, followed by saasCNV and ending with CNVkit, which carries the lowest values (Figure S5). Even though all GI parameters followed this hierarchy between pipelines, three differences stood out over the others.

CNVkit had worse detection performance of losses in favor of LOHs, probably caused by the analysis methodology to identify this type of alterations, since the baseline to detect heterozygosity loci was established with a panel of samples. On the other hand, in the case of saasCNV, difficulties appeared in the identification of gain events. Here, there was hardly any gain event detected, showing a weakness of the pipeline. Lastly, SureCall presented a different distribution of LOH events according to size. Whereas saasCNV and CNVkit showed a higher number of LOH larger than 15 mb, SureCall presented more LOH events above 15 Mb, having an opposite distribution in size.

Since an objective and robust technical benchmark was required to compare studied pipelines, the accuracy to determine GI patterns was measured according to clinical outcome, particularly PFI, to endow our findings with clinical significance. Hence, the correlation between each GI parameter from different pipelines and response to platinum was obtained. saasCNV pipeline showed the lowest statistical power in all the evaluated parameters. The total number of LOH events (p=0.0081) presented the highest signification, however, CNVkit also headed in this regard. On the contrary, SureCall and CNVkit presented higher statistical significance than saasCNV, each one presenting a correlation with different GI parameters. While

CNVkit showed a higher correlation when comparing PFI with global GI (total number of events and percentage of genome altered) as well as LOH events, SureCall presented higher accuracy with parameters related to gains and losses (Supplementary File 1). In all cases, higher number of CN events, as well as altered genome, correlated with longer responses to platinum-based chemotherapy (Supplementary file 1).

Finally, CNVkit was chosen according to best performance, higher number of customizable parameters, advantageous use of on-target and off-target read counts, widening the coverage of the panel, and suitability to medium size NGS panels. Hence, following analysis to perform and adjust the predictive model were done using CNVkit pipeline.

Supplementary data S2.- Methods model fitting: Three strategies were used to fit the model: raw read counts, GI parameters derived from CNVkit algorithm and HTG gene expression panel data. As the outcome, a clinical dichotomic variable was used: less than 12 months or equal or more than 12 months of PFI.

For the first approach, raw read counts from 170080 Single Nucleotide polymorphisms (SNPs) contained in the Oneseq backbone and Custom panel were used to feed the model. These SNPs are uniformly distributed along the entire genome at a

resolution of 1 Mb. To reduce the dimensionality of the data frame, different strategies were applied: SNPs counts were selected by sorting the p-values obtained from an Anova test, selecting those with the highest signal-to-noise (s2n) ratio (the quotient between the subtraction from the mean of the groups and the sum of its variances); the significant discriminant SNPs, using the principal components and in a binomial logistic regression regularized with elastic net.

After feature selection, chosen SNPs were adjusted with Support Vector Machine (SVM) applying radial kernel, Random Forest (RF) or Neural Network (NN) algorithms, performing hyperparameters tuning as follows: a) In SVM the assayed values of the penalty factor for margins violation (cost, C) were 1, 10, 50, 100, 500, 700, 1000, 1500, whereas Gaussian Width, represented by Sigma in the kernel of SVM equation, takes values of 0.0001, 0.001, 0.01; b) In the RF algorithm, the considered number of variables randomly selected at each split (mtry) were 2, 5, 10 and 50. Otherwise, the minimal sizes of the nodes were 2, 3, 4, 5 and 10. And the used split rule was Gini index; c) In the NN tuning, sizes of 5, 10, 15, 20, 40 layers and values of 0.01 and 0.1 in decay in the weights of the loss function were considered.

The second approach applies GI parameters derived from CNVKit algorithm. These parameters are based on events of gains, losses and Loss of Heterozygosity (LOH) (Supplementary Data 1). Due to the lower number of parameters, this approach presents a less stringent feature selection, simplifying the filtering strategy. Parameters derived from a decision tree were curated by an expert in the field with the aim of endowing the dataset with clinical significance. The debugged data frame comprised 30 variables.

The third strategy assesses the expression of 2459 genes included in the HTG-OBP. Feature and algorithms selection were analogous to the SNPs scheme. Logistic regression was excluded as a feature selection method considering that only two genes presented discriminant power.

Supplementary data S3.- Selected parameters:

In the first layer, the following 8 SNPs were selected: rs876261, rs142099227, rs13135475, rs56747986, rs540649069, rs761256207, rs13401599, rs562439697 (Table S2).

The GI layer comprises the 28 parameters: Number of events, Mb of altered genome, Percentage of altered genome, Number of events excluding copy numbers between 0.5 and 3, Mb of altered genome excluding copy numbers between 0.5 and 3, Percentage of genome altered excluding copy numbers between 0.5 and 3, Total number of

gain events, Mb of genome altered by gains, Percentage of genome altered by gains, total number of gain events, Mb of genome altered by gains and percentage of genome altered by gains between 0.5 and 3, total number of loss events, Mb of genome altered by losses, Percentage of genome altered by losses, Total number of LOH events, Mb of genome altered by LOHs, Percentage of genome altered by LOHs, Total number of LOH>15 Mb, Mb of genome altered by LOHs>15 Mb, Percentage of genome altered by LOHs>15 Mb, Total number of LOH>10 Mb, Mb of genome altered by LOHs>10 Mb, Percentage of genome altered by LOHs>10 Mb and Loss of Heterozygosity (LOH) Long-Scale Transition (LST), Tellomeric Allelic Imbalance (TAI) and scoreHRD from scarHRD package.

Whereas the transcriptomic HTG model is fed with *RUVBL1, ADORA2A, ABCD4, PVR, PFDN2, SIL1, FGF11* expression (Table S3).

Supplementary Figures

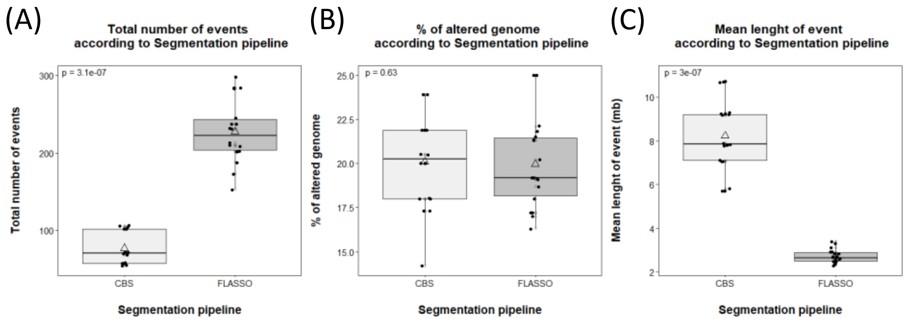

Figure S1: Differences in GI parameters according segmentation used in CNVkit pipeline. **(A)** Total number of events. **(B)** Percentage of altered genome. **(C)** Median length of event

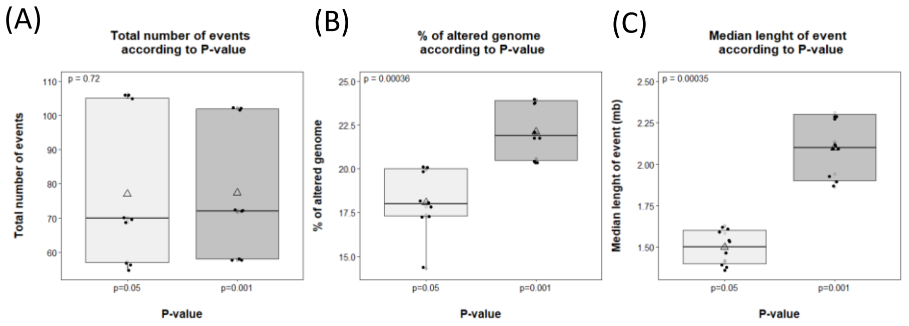

Figure S2: Differences in GI parameters adjusting p-value in CNVkit pipeline. **(A)** Total number of events. **(B)** Percentage of altered genome. **(C)** median length of event

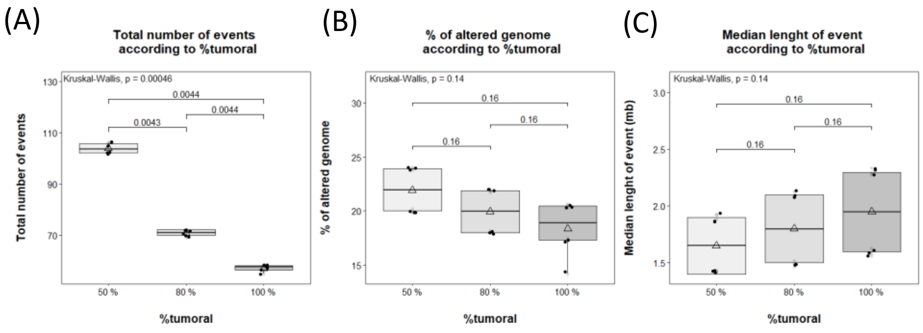

Figure S3: Differences in GI parameters according tumor burden in CNVkit pipeline. **(A)** Total number of events. **(B)** Percentage of altered genome. **(C)** median length of event

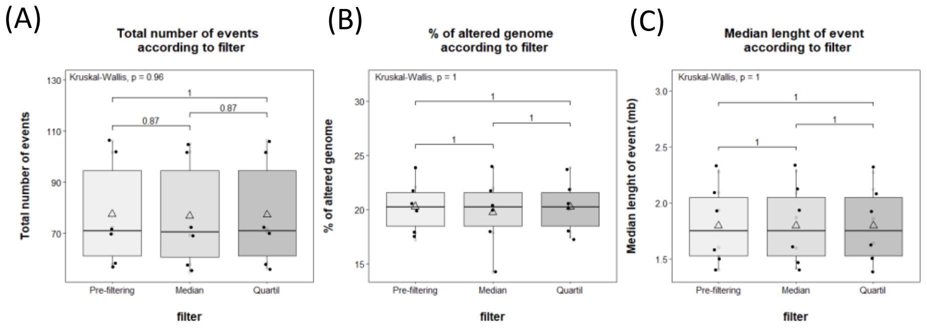

Figure S4: GI parameters according to pre-filtering step in saasCNV pipeline. **(A)** Total number of events. **(B)** Percentage of altered genome. **(C)** Median length of event

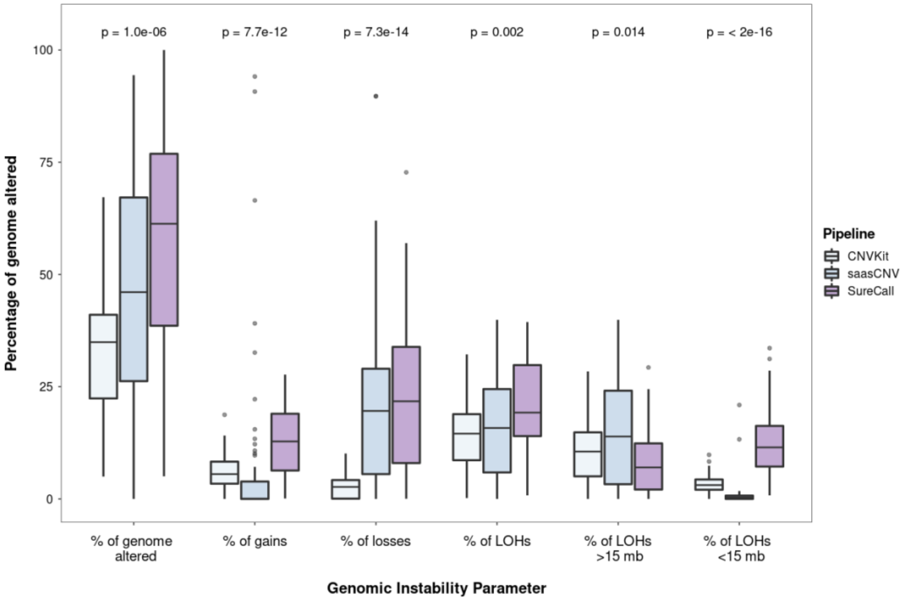

Figure S5. Comparison of GI parameters between implemented pipelines, highlighting differences for the assessment of each feature.

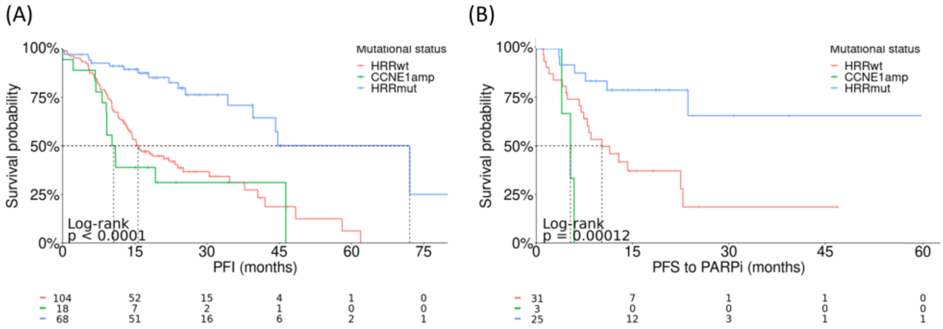

Figure S6. Log-Rank test to evaluate the predictive information of HRR-gene mutation and CCNE1 amplification-based classification regarding (A) PFI and (B) PFS to PARPi.

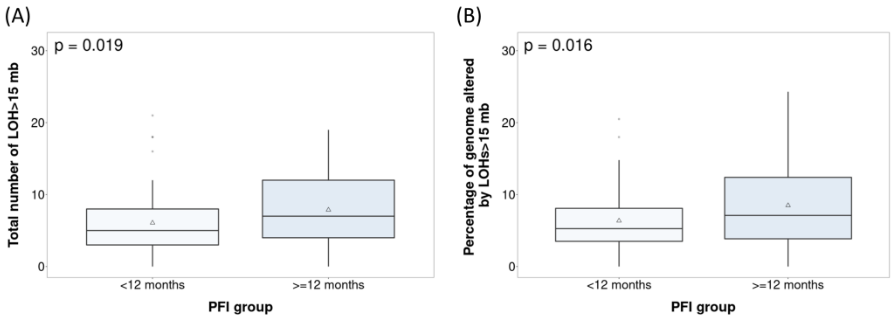

Figure S7. Correlation of GI parameters with PFI assessed by non-parametric tests. **(A)** Boxplot showing the distribution of the number of LOH events bigger than 15 Mb between PFI-based populations; **(B)** Boxplot showing the percentage of genome altered by LOH events bigger than 15 Mb between PFI-based populations.

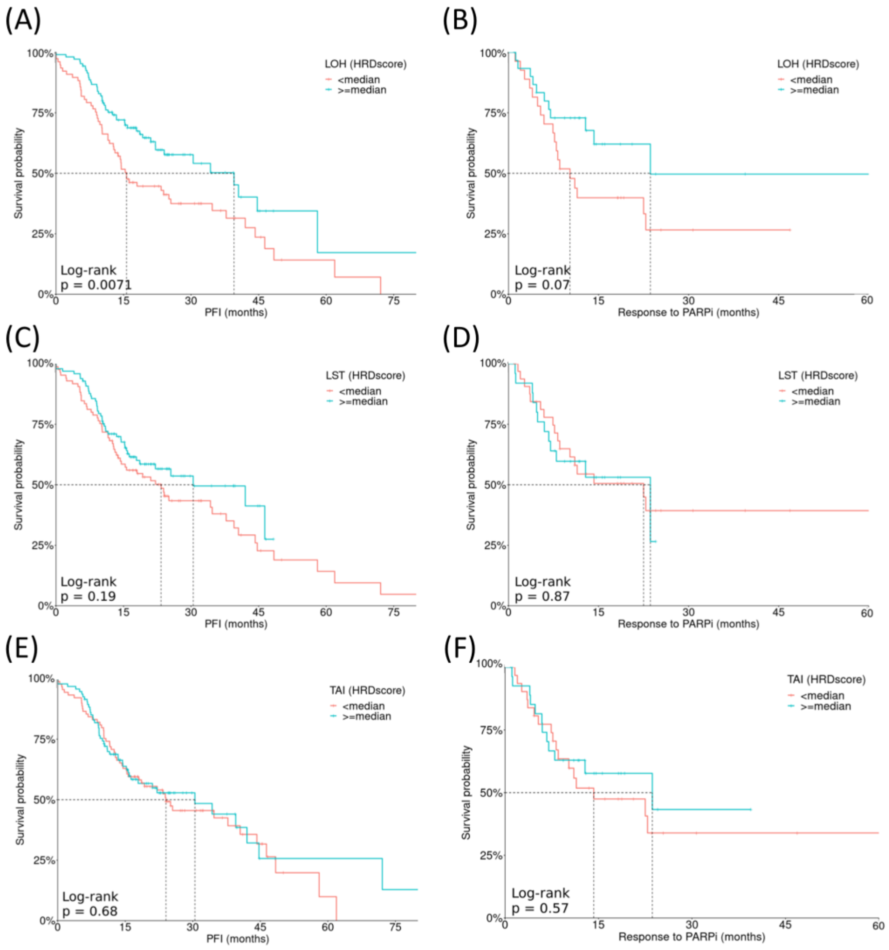

Figure S8. Log-Rank tests evaluating the implication of predefined HRD scars parameters from scarHRD package in correlation with PFI and PARPi response. Log-rank tests evaluating performance of: **(A)** LOH, **(C)** LST and **(E)** TAI versus PFI and **(B)** LOH, **(D)** LST and **(F)** TAI performance versus PARPi response.

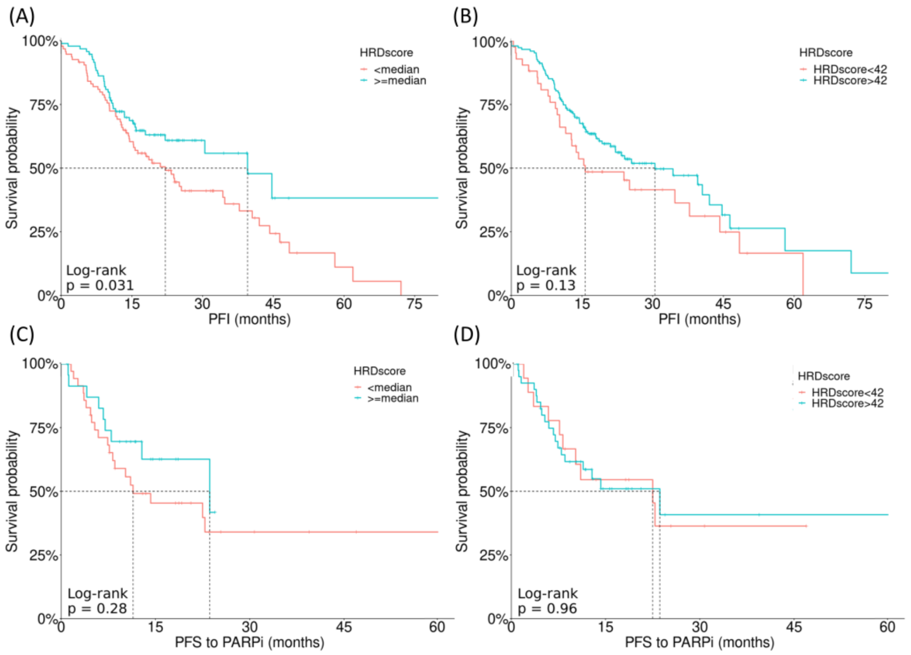

Figure S9. Correlation of HRD score obtained on scarHRD package and time-to-event variables. Log-rank tests evaluating: **(A)** Platinum free interval prediction regarding HRD score median-based stratification. **(B)** Pre-established Myriad-based cut-off stratification (42). **(C)** PFS to PARPi prediction regarding HRD score median-based stratification. **(D)** Pre-established Myriad-based cut-off stratification (42).

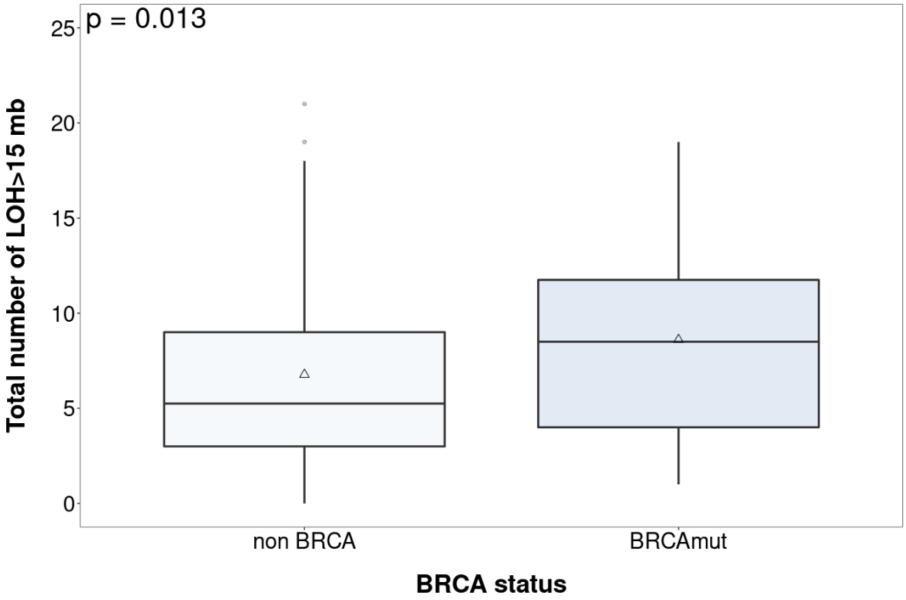

Figure S10. Distribution of total number of LOH events > 15 Mb between BRCA-based populations.

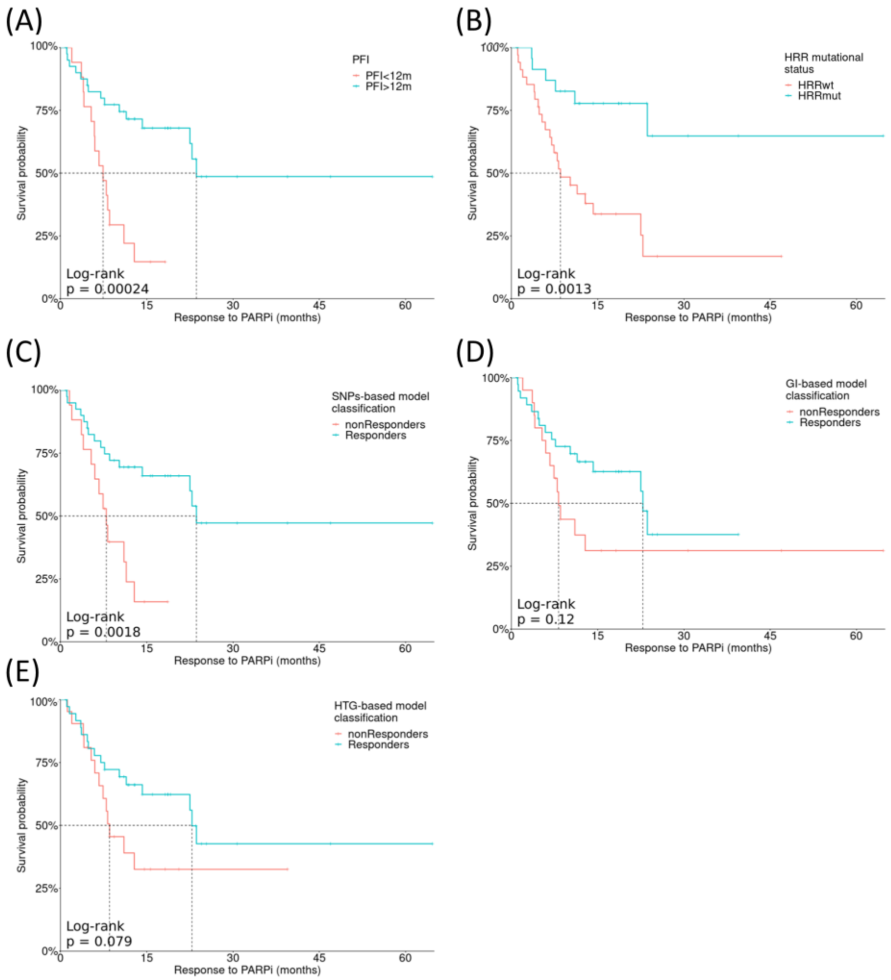

Figure S11. Log-Rank tests evaluating the implication of predictive models with PARPi response. **(A)** PFI stratification, **(B)** HRR mutation, **(C)** SNP-based model, **(D)** GI-based model and **(E)** HTG-based model.

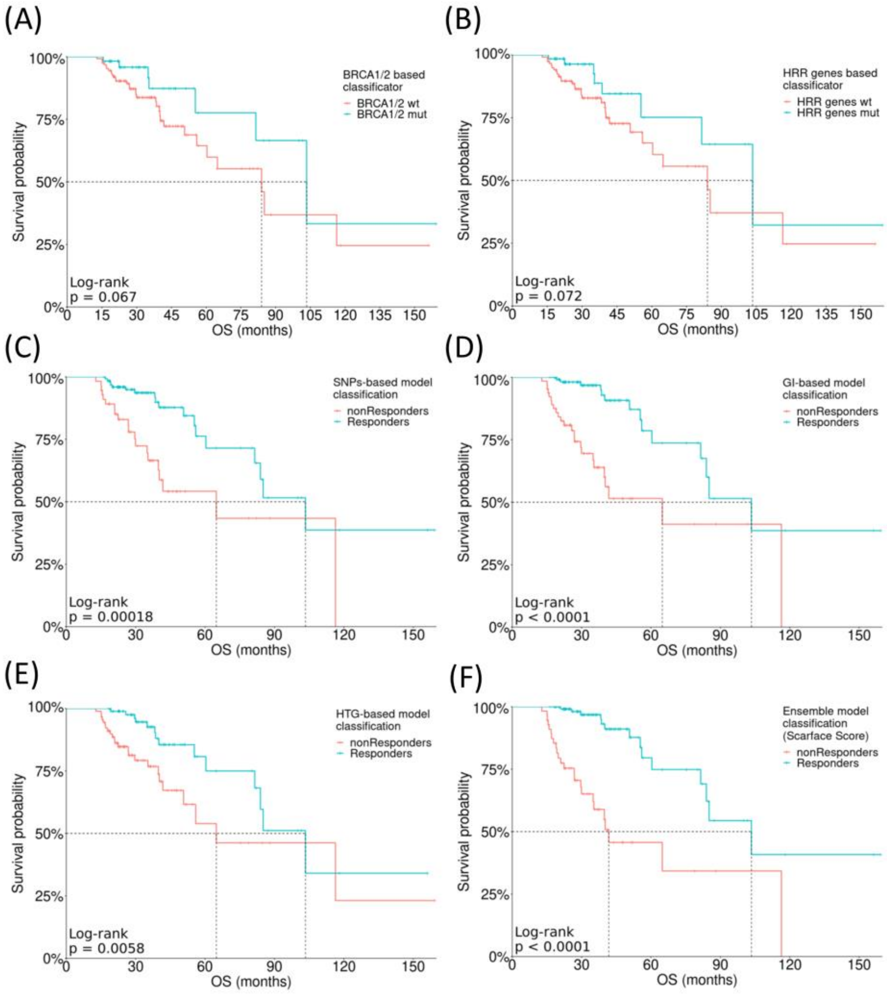

Figure S12. Log-Rank tests evaluating the implication of mutational-based classifiers and predictive models with OS. **(A)** BRCA1/2-based classifier **(B)** HRR-based classifier **(C)** SNP-based model, **(D)** GI-based model, **(E)** HTG-based model and **(F)** Ensemble model.

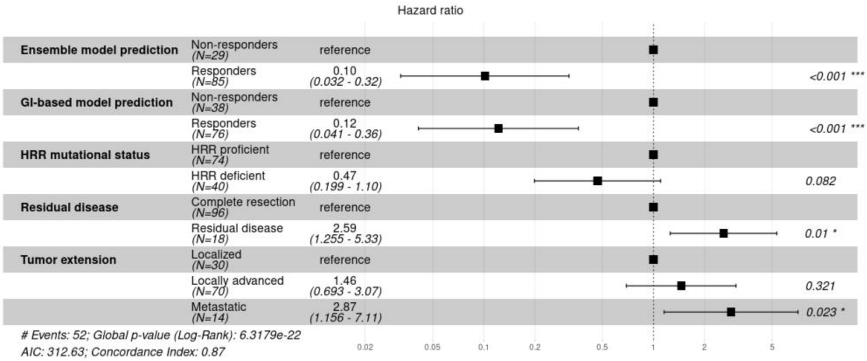

Figure S13: Multivariate analysis performed by Cox regression for clinicopahological parameters, HRR alteration and three-source model performance in addition to ensemble model. Tumor extension was stratified based on stage; localized (I-IIB), locally advanced (III-IVA) and metastatic (IVB).

Supplementary Tables

Table S1: Selected parameters SNPs model

**SNP**	**Chr**	**Position** (GRCh38)	**Alleles**	**Consequence**	**Clinical significance**
rs876261	chr8	95230391	C>G	None	Not Reported in ClinVar
rs142099227	chrx	102701669	C>A	ARMCX5-GPRASP2 : Intron Variant	Not Reported in ClinVar
rs13135475	chr4	39498624	T>C / T>G	UGDH : 500B Downstream Variant	Not Reported in ClinVar
rs56747986	chr13	97118261	T>A	None	Not Reported in ClinVar
rs540649069	chr4	97223716	T>G	STPG2 : Intron Variant	Not Reported in ClinVar
rs761256207	chrx	70504827	G>A	DLG3 : 3 Prime UTR Variant	Not Reported in ClinVar
rs13401599	chr2	834796	A>G / A>T	LINC01115 : Intron	Not Reported

				Variant	in ClinVar
rs562439697	chr3	14679706	G>A / G>T	C3orf20 : Intron Variant	Not Reported in ClinVar

Table S2: Selected parameters HTG model

**Gene**	**Location**	**Cytogenetic band**	**Size (bases)**	**Function**
**RUVBL1**	chr3:128,064,611-128,153,914	3q21.3	89,304	Activity ATPase associated with diverse cellular activities.
**ADORA2A**	chr22:24,417,879-24,442,357	22q11.23	24,479	Guanine nucleotide-binding protein (G protein)-coupled receptor (GPCR).
**ABCD4**	chr14:74,285,269-74,303,062	14q24.3	17,794	ATP-binding cassette (ABC) transporters. ABC proteins

				transport various molecules across extra- and intra-cellular membranes.
**PVR**	chr19:44,643,798-44,666,162	19q13.31	22,365	Transmembrane glycoprotein belonging to the immunoglobulin superfamily.
**PFDN2**	chr1:161,100,556-161,118,055	1q23.3	17,500	The encoded protein is one of six subunits of prefoldin, which is a chaperone protein that binds and stabilizes newly synthesized polypeptides.

**SIL1**	chr5:138,946,724-139,293,557	5q31.2	346,834	This gene encodes a N-linked glycoprotein with an N-terminal ER targeting sequence, 2 putative N-glycosylation sites, and a C-terminal ER retention signal.
**FGF11**	chr17:7,341,617-7,348,256	17p13.1	6,640	The protein encoded by this gene is a member of the fibroblast growth factor (FGF) family

Table S3. Weights and main characteristics of the parameters included in the layer 1 of SNPs deep sequencing

Chromosome	Start genomic position	End genomic position	Rs reference	Weight
8	96242618	96242619	rs876261	-8.542673
X	101956596	101956597	rs142099227	20.0047
4	39500243	39500244	rs13135475	21.22615
13	97770514	97770515	rs56747986	10.21631
4	98144866	98144867	rs540649069	-5.365423
X	69724676	69724677	rs761256207	-3.96197
2	830764	830765	rs13401599	16.8416
3	14721212	14721213	rs562439697	11.81417

Table S4. Weights and main characteristics of the parameters included in the layer 2 of Genomic Instability related parameters

**Parameter**	**Weight**
Total Events of Copy Number Alteration (CNA)	-89.32514
Megabases (Mb) altered with CNA	-40.40662
Percentage of altered genome	-40.40662
Total events excluding those carrying a log2Ratio of the CNAs 0.3 and 0.5 and without Copy Number of 3	21.87199
Mb altered excluding those carrying a log2Ratio of the CNAs 0.3 and 0.5 and without Copy Number of 3	-12.187
Percentage of genome altered excluding this carrying a log2Ratio of the CNAs 0.3 and 0.5 and without Copy Number of 3	12.18277
Total gain events	-109.3775
Mb affected by gain phenomenon	-107.0544
Percentage of genome affected by gain phenomenon	-107.054

Total gain events excluding those with CN of 3	-336.0565
Mb affected by gain phenomenon excluding those with CN of 3	-5.217328
Percentage of genome affected by gain phenomenon excluding those with CN of 3	-5.214158
Total loss events	-40.81254
Mb affected by loss phenomenon	84.78166
Percentage of genome affected by loss phenomenon	84.77692
Total Loss of Heterozigosity (LOH) events	-60.84025
Mb affected by LOH phenomenon	18.99282
Percentage of genome affected by LOH phenomenon	18.99464
LOH events longer than 15 Mb	-8.25084
Mb affected by LOH events longer than 15 Mb	47.53902
Percentage of genome affected by LOH	47.54521

events longer than 15 Mb	
Events of LOH events longer than 10 Mb	-88.37638
Percentage of genome affected by LOH events longer than 10 Mb	60.30851
Mb of genome affected by events of LOH longer than 10 Mb	60.3130
LOH score inferred by scarHRD algorithm	20.74359
Telomere Allelic Imbalance (TAI) inferred by scarHRD algorithm	-17.81501
Large Scale Transition (LST) inferred by scarHRD algorithm	-46.20552
HRD score inferred by scarHRD algorithm	-40.28818

Table S5. Weights and main characteristics of the parameters includ-ed in the layer 3 of gene expression

Parameter	Value
b→h1	0.32114585
RUVBL1→h1	1.46186113
ADORA2A→h1	1.13517002
ABCD4->h1	-1.42001647
PVR->h1	3.6030025
PFDN2→h1	-3.5589472
SIL1→h1	-0.07240374
FGF11->h1	2.50794528
b->h2	0.11839882
RUVBL1->h2	-4.14981406
ADORA2A->h2	1.56677479
ABCD4->h2	0.65593751
PVR->h2	3.70235402
PFDN2->h2	3.20518767
SIL1->h2	-2.9256243
FGF11->h2	-1.69884204
b->h3	2.15686463
RUVBL1->h3	0.76914432

ADORA2A->h3	0.20556746
ABCD4->h3	4.69695949
PVR->h3	-0.93690447
PFDN2->h3	-0.77557672
SIL1→h3	-1.91350636
FGF11->h3	-2.79761149
b->h4	0.6009593
RUVBL1->h4	0.80754756
ADORA2A->h4	1.34044963
ABCD4→h4	0.68130962
PVR->h4	-3.58710134
PFDN2->h4	-2.01115627
SIL1->h4	2.34581033
FGF11->h4	0.92617267
b->h5	-0.28114186
RUVBL1→h5	-2.28981767
ADORA2A->h5	2.53377303
ABCD4->h5	4.8764124
PVR→h5	-1.15424853
PFDN2->h5	-0.36806824
SIL1->h5	-1.32683523

FGF11->h5	-1.05912051
b->o	-5.33788387
h1->o	3.27416548
h2->o	3.94796929
h3->o	7.2357045
h4->o	7.88059076
h5->o	-7.08780599

Table S6. Weights and main characteristics of the parameters included in the lensemble scarface model

Chromosome (SNPs) / Parameter	Start genomic position	End genomic position	Rs reference	Weight
8	96242618	96242619	rs876261	1.797972
X	101956596	101956597	rs142099227	-14.41997
4	39500243	39500244	rs13135475	3.44995
13	97770514	97770515	rs56747986	-11.80795
4	98144866	98144867	rs540649069	-7.941518
X	69724676	69724677	rs761256207	-13.1249
2	830764	830765	rs13401599	-10.39273
3	14721212	14721213	rs562439697	-4.478431
Total Events of Copy Number Alteration (CNA)				-0.3446789
Megabases (Mb) altered with CNA				3.933628
Percentage of altered genome				3.933628

Total events excluding those carrying a log2Ratio of the CNAs 0.3 and 0.5 and without Copy Number of 3				0.4648378
Mb altered excluding those carrying a log2Ratio of the CNAs 0.3 and 0.5 and without Copy Number of 3				3.258229
Percentage of genome altered excluding this carrying a log2Ratio of the CNAs 0.3 and 0.5 and without Copy Number of 3				3.258231
Total gain events				-2.274835
Mb affected by				2.382054

gain phenomenon				
Percentage of genome affected by gain phenomenon				2.382074
Total gain events excluding those with CN of 3				-9.462503
Mb affected by gain phenomenon excluding those with CN of 3				-7.862805
Percentage of genome affected by gain phenomenon excluding those with CN of 3				-7.862848
Total loss events				4.138344
Mb affected by loss phenomenon				7.051245
Percentage of genome affected by				7.051268

loss phenomenon				
Total Loss of Heterozigosity (LOH) events				-0.9454972
Mb affected by LOH phenomenon				2.281842
Percentage of genome affected by LOH phenomenon				2.281836
LOH events longer than 15 Mb				-0.6856251
Mb affected by LOH events longer than 15 Mb				2.683343
Percentage of genome affected by LOH events longer than 15 Mb				2.683316
Events of LOH events longer than 10 Mb				0.4925579

Percentage of genome affected by LOH events longer than 10 Mb				2.907711
Mb of genome affected by events of LOH longer than 10 Mb				2.907722
LOH score inferred by scarHRD algorithm				-16.48205
Telomere Allelic Imbalance (TAI) inferred by scarHRD algorithm				-17.36565
Large Scale Transition (LST) inferred by scarHRD algorithm				-13.00467
HRD score inferred by scarHRD algo-				-18.11746

rithm				
VTCN1				17.55631
PTCRA				2.636084
LRP2				-18.58385
NLK				-4.829101
DLL3				-13.52395
SLC22A6				1.858745
VPS13A				-1.32965

Table S7. Log-rank test results for single-source and ensemble model. Additionally, PFI, BRCA (germline and somatic mutations) and HRR-based (all HR-genes interrogated in the panel, including BRCA1/2) classifications were added.

	Model	N	events	Median (CI 95%)	P-value
PFI	*BRCA1/2* mutation	127	83	15.7(13.4-23.9)	$3x10^{-6}$
		56	16	72.1(44.2-NA)	
	HRR mutation	119	82	15.2(12.5-22.1)	$6x10^{-8}$
		64	17	44.7(39.5-NA)	
	SNP-based	56	45	9.43(7.23-11.0)	$<2x10^{-16}$
		127	54	39.53(34.3-48.4)	
	GI-based	65	56	9.07(7.17-10.2)	$<2x10^{-16}$
		118	43	42.03(37.8-NA)	
	HTG-based	91	59	11(9.93-23.3)	$1x10^{-7}$
		92	40	42(34.7-61.9)	
	Ensemble	56	53	7.8(6.63-9.43)	$<2x10^{-16}$
		127	46	42.0(37.8-61.90)	
PARP	PFI 12 months	17	14	7.4(5.9-NA)	0.00024
		41	15	23.6(22.5-NA)	
	BRCA1/ mutation	37	23	10.2(7.5-NA)	0.0048
		21	6	NA (23.6-NA)	
	HRR mutation	34	23	8.53(6.7-NA)	0.0013
		24	6	NA(23.6-NA)	
	SNP-based	17	13	7.97(5.97-NA)	0.0018
		41	16	23.63(22.5-NA)	

	**GI-based**	20	13	8.2(6.7-NA)	0.12
		38	16	22.9(14.2-NA)	
	**HTG-based**	21	13	8.53(6.7-NA)	0.08
		37	16	22.87(14.2-NA)	
	**Ensemble**	16	13	7.68(5.33-NA)	0.00077
		42	16	23.63(22.50-NA)	
**OS**	***BRCA1/2* mutation**	127	31	83.9(60.4-NA)	0.0067
		56	7	103.4(81.5-NA)	
	**HRR mutation**	19	30	83.9(60.4-NA)	0.072
		64	8	103.4(81.5-NA)	
	**SNP-based**	56	20	64.9(39.8-NA)	0.00018
		127	18	103.4(81.5-NA)	
	**GI-based**	65	24	64.9(39.8-NA)	$3x10^{-6}$
		118	14	103.4(83.9-NA)	
	**HTG-based**	91	24	64.9(50.7-NA)	0.0058
		92	14	103.4(83.9-NA)	
	**Ensemble**	56	24	41.7(35.0-NA)	$1x10^{-8}$
		127	14	103.4(83.4-NA)	
